# Rare case of left epididymo-orchitis complicated by pampiniform plexus thrombosis: A case report

**DOI:** 10.1016/j.radcr.2025.02.074

**Published:** 2025-03-15

**Authors:** Hasan N Al-Haidari, Raad Y Altahat, Khaled Abu Jamous, Mohammad F Hamidi, Ibrahim K Alhmaisat, Amira H Alquati

**Affiliations:** a Radiology Department, Jordanian Royal Medical Services, King Hussien Medical Center, Amman, Jordan; b Radiology Department, Alsaudi Hospital, Amman, Jordan; c Urology Department, Alsaudi Hospital, Amman, Jordan; d Radiology Department, Ministry of Health, Al-Basheer Hospital, Amman, Jordan

**Keywords:** Epididymo-orchitis, Thrombosis, Pampiniform plexus thrombosis, Varicocele, Ultrasound

## Abstract

Acute scrotal pain includes urgent conditions in urology, such as testicular torsion, testicular rupture, epididymo-orchitis, and abscess. However, varicocele (pampiniform plexus) thrombosis is considered to be a rare cause of such pain. We herein report a case of a 27-year-old male patient with a history of epididymo-orchitis, who complained of painful scrotal swelling. Ultrasonography showed left-side pampiniform plexus thrombosis. This case highlights a rare condition, which should be included in the differential diagnosis of acute scrotal pain, indicating the need for further studies to elucidate its pathophysiology and provide proper treatment for such cases.

## Introduction

Acute scrotal pain is a urological emergency; on the other hand, pampiniform plexus thrombosis is considered an extremely rare cause of acute scrotal pain. There are several possible predisposing factors for thrombosis, such as vascular endothelial injuries, sluggish venous flow, and hypercoagulability; however, infection has not been reported as the main factor. A few cases of thrombosed varicocele have been reported in patients with epididymo-orchitis. Alshubaili et al. [1] published the first case in 2020. A second case was reported in 2021 by Hamdouni et al. [2].

To our knowledge, this is the third case; however, it was on the left side**,** which is the more common side for varicocele rather than on the right side, as in the previous 2 cases.

## Case report

**Fig. 1:**
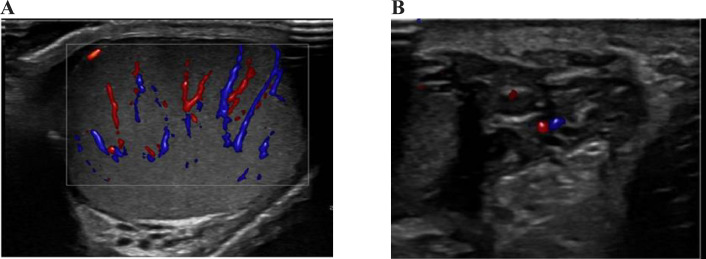
Epidedymoorchitis. (A) Shows increased vascularity of the testis. (B) Shows echogenicity of the epididymis. Note the varicocele on both images with intrascrotal extension in A.

**Fig. 2:**
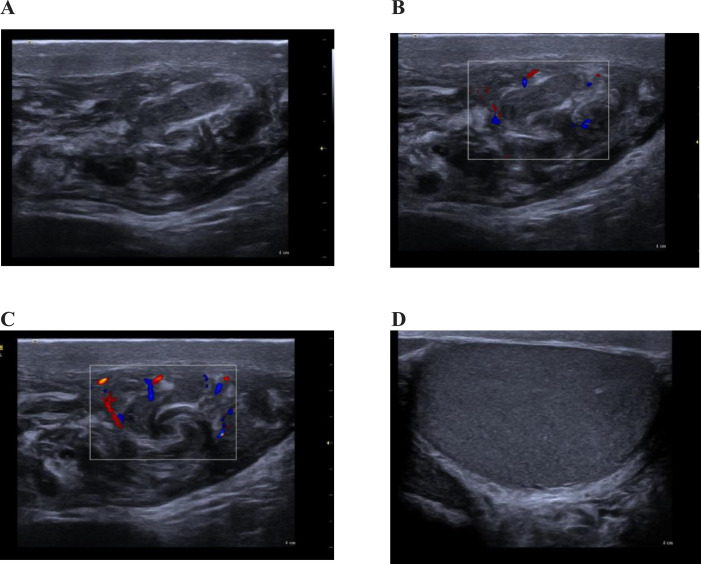
Left thrombosed varicocele (2 weeks after). (A) Shows dilated pampiniform plexuses with echogenic content. (B and C) Show no intraluminal Doppler flow noted the enlarged heterogenous epididymis due to epidedymitis. (D) Shows normal left testis with intrascrotal extension of the varicocele.

A 27-year-old single male patient, presented to the emergency department with a complaint of painful swelling in the left hemiscrotum for a few hours; the patient did not report any other symptoms. The patient was diagnosed with left epididymo-orchitis (Fig. 1) and was started on oral antibiotics and analgesics for approximately 2 weeks. The patient had no significant medical or surgical history. Physical examination revealed swelling and tenderness in the left scrotum with no overlying skin changes. The laboratory results were unremarkable. The complete blood count was normal (erythrocytes: 5.28 × 10^12^/L, white blood cells: 8.1 × 10^9^/ L [neutrophils 89%, lymphocytes 8%, monocytes 5%, eosinophils and basophils, 0%]). Scrotal ultrasonography was performed to rule out a strangulated inguinal hernia or abscess formation. Ultrasound revealed slight heterogeneity of the left testis of a normal size with an enlarged, heterogeneous left epididymis. In addition, dilated pampiniform plexuses with intraluminal echogenic content were observed, with no Doppler flow or pulse waves (Fig. 2). The diagnosis based on imaging was thrombosed varicocele, and the patient was urgently referred to the urology team for further evaluation and treated surgically (varicocelectomy).

## Discussion

A varicocele is an abnormal dilatation of the scrotal venous pampiniform plexus, which drains blood from each testicle into the respective testicular veins [3]. It is more common on the left side because of the anatomical orientation of the left testicular vein, which is more perpendicular in its course and joins the relatively high-pressure left renal vein [4]. Varicocele thrombosis is a rare condition that may occur spontaneously or postoperatively. In their case report of spontaneous pampiniform plexus thrombosis, Kamal et al. [5] stated that only 20 cases of this uncommon condition had been documented in the literature.

Epididymo-orchitis is considered a major cause of acute scrotal pain [6]. It may cause severe testicular necrosis, atrophy, infarction, and formation of scrotal abscesses [7]. Previous studies have not recognized infection as a predisposing factor for thrombosis of the testicular vein; however, it may have been a contributing factor in our case, owing to the lack of other factors.

Although immunothrombosis, or inflammation-induced thrombosis, is a well-known condition and an emergency host mechanism that contains infections at the site of entry and prevents spread. However, excessive and uncontrollable immunothrombosis, also known as microvascular thrombosis, causes significant organ damage via thromboinflammation. Typical manifestations include stroke, myocardial infarction, and venous thromboembolism. There are complex interactions between haemostasis and inflammation through numerous factors, such as proinflammatory cytokines, chemokines, adhesion molecules, tissue factor expression, platelet and endothelial activation, and microparticles. Thrombosis is caused by inflammation that increases procoagulant factors and impairs natural anticoagulant mechanisms and fibrinolytic activity, resulting in a thrombotic tendency. Moreover, chronic inflammation may cause endothelial damage, which can result in the loss of the physiological anticoagulant, antiplatelet, and vasodilatory properties of the endothelium. Conversely, inflammation-induced venous thrombosis can occur even in the absence of endothelial damage. On the other side, coagulation also augments inflammation creating a vicious cycle. This is primarily achieved through the thrombin-induced release of proinflammatory cytokines and growth factors. Platelets may activate dendritic cells, which in turn induce inflammation [8,9].

The association between inflammation and thrombosis is considered one of the main causes of varicocele thrombosis. This relationship also demonstrates how the majority of cases are treated conservatively and that anti-inflammatory approaches might help fight thrombotic complications in infectious diseases. For example, Gleeson et al. considered the use of nonsteroidal anti-inflammatory agents as a sufficient management option without the need for anticoagulants [10]. Overall, pain management using nonsteroidal anti-inflammatory agents may be sufficient to manage these cases after treating the primary cause of thrombosis [2].

## Conclusion

A thrombosed varicocele is an uncommon cause of acute testicular pain. Several factors have been identified as causes of thrombosed varicocele, with epididymo-orchitis considered a primary cause. This entity is poorly described in the literature, and available data are limited to a few cases; therefore, further evaluation and research should be conducted to establish the pathophysiology and appropriate treatment for such cases.

## Patient consent

Written informed consent was obtained from the patient for the publication of this case report and any accompanying images. The patient was informed that no identifying information would be disclosed.
